# Evidence for PMAT- and OCT-like biogenic amine transporters in a probiotic strain of *Lactobacillus*: Implications for interkingdom communication within the microbiota-gut-brain axis

**DOI:** 10.1371/journal.pone.0191037

**Published:** 2018-01-11

**Authors:** Mark Lyte, David R. Brown

**Affiliations:** 1 Department of Veterinary Microbiology and Preventive Medicine, College of Veterinary Medicine, Iowa State University, Ames, Iowa, United States of America; 2 Department of Veterinary and Biomedical Sciences, College of Veterinary Medicine, University of Minnesota, St. Paul, Minnesota, United States of America; Okayama Daigaku, JAPAN

## Abstract

The ability of prokaryotic microbes to produce and respond to neurochemicals that are more often associated with eukaryotic systems is increasingly recognized through the concept of microbial endocrinology. Most studies have described the phenomena of neurochemical production by bacteria, but there remains an incomplete understanding of the mechanisms by which microbe- or host-derived neuroactive substances can be recognized by bacteria. Based on the evolutionary origins of eukaryotic solute carrier transporters, we hypothesized that bacteria may possess an analogous uptake function for neuroactive biogenic amines. Using specific fluorescence-based assays, *Lactobacillus salivarius* biofilms appear to express both plasma membrane monoamine transporter (PMAT)- and organic cation transporter (OCT)-like uptake of transporter-specific fluorophores. This phenomenon is not distributed throughout the genus *Lactobacillus* as *L*. *rhamnosus* biofilms did not take up these fluorophores. PMAT probe uptake into *L*. *salivarius* biofilms was attenuated by the protonophore CCCP, the cation transport inhibitor decynium-22, and the natural substrates norepinephrine, serotonin and fluoxetine. These results provide the first evidence, to our knowledge, for the existence of PMAT- and OCT-like uptake systems in a bacterium. They also suggest the existence of a hitherto unrecognized mechanism by which a probiotic bacterium may interact with host signals and may provide a means to examine microbial endocrinology-based interactions in health and disease that are part of the larger microbiota-gut-brain axis.

## Introduction

Bacteria entering the lumen of the intestinal tract encounter a plethora of biologically-active substances present in luminal fluids. Among these molecules are the primary amines norepinephrine (NE), dopamine, histamine, tyramine and serotonin (SER), which are synthesized and function as paracrine mediators and neurotransmitters within animal hosts [1–3]. In a large and growing number of studies, the neurotransmitter NE in particular has been shown to act directly on many bacterial species to enhance their growth and virulence characteristics [4–6]. Tyramine, a chemically-related trace amine, also influences bacterial growth and interactions with intestinal epithelial cells [7, 8]. Some bacterial species, notably *Lactobacilli*, are known to synthesize biogenic amines that can interact with trace amine receptors and thereby affect their animal hosts, including agmatine, histamine, ß-phenylethylamine, and tyramine [9]. Furthermore, enteric microbes have recently been shown to modulate serotonin concentrations in gut luminal fluid [10].

Biogenic amines such as NE, dopamine and serotonin are taken up by mammalian cells through plasma membrane cation transporters, which are members of a large family of solute carrier (SLC) transport proteins [11]. Specific transport proteins for these neurotransmitter substances are members of the *SLC6* gene family and are expressed on neural membranes where they mediate Na^+^-dependent transmitter uptake from synaptic zones by a process referred to as “uptake-1”. A second, unrelated group of transport proteins are expressed in non-neuronal cells, which take up extracellular biogenic amines and other cationic substances through an uptake-2 process. Analogs of some of these latter eukaryotic transporters are expressed in prokaryotes. Analogous bacterial glutamate and leucine transporters have been discovered that have informed studies of the structure-function relationships of eukaryotic SLC transporters [12, 13]. This raises the question of whether bacteria residing in the gut lumen express uptake systems that would permit them to acquire and accumulate neuroactive serotonin, NE or related transmitter substances. It has been hypothesized that probiotic bacteria may act as carriers of these substances [14].

To test this hypothesis, we examined monoamine uptake into *Lactobacillus* biofilms using fluorescent probes for membrane amine transport. Numerous studies have provided evidence that biofilm-forming lactobacilli have multiple beneficial effects on digestive health [15]. The cationic fluorescent probes IDT307 and ASP^+^ were used as well-defined substrates for membrane-associated “uptake-2” amine transporters in mammalian cells [16–18]. IDT307 ((4-(4-(dimethylamino)phenyl)-1-methylpyridinium iodide) is a fluorescent derivative of 1-methyl-1-phenylpyridinium, a specific substrate for the uptake-2 plasma membrane monoamine transporter (PMAT) encoded on the *SLC29A4* gene [19]. IDT307 has recently been used to screen potential PMAT inhibitors, including those which suppress serotonin uptake through the low affinity, high capacity serotonin transporter, the molecular target of fluoxetine and other common antidepressant drugs [18]. The chemically-related fluorescent cationic substance, ASP^+^ (4-(4-(dimethylamino)styryl)-N-methylpyridinium iodide), has been used as a specific substrate for the three defined organic cation transporters (OCTs) encoded in the *SLC22* gene family [20]. These latter polyspecific transporters mediate the uptake of cationic drugs, endogenous amines and other substrates into neurons and other mammalian cell types. The results of the present study suggest that some *Lactobacillus* biofilms express functional homologs of PMAT and OCTs, which could take up and potentially deliver bioactive amines to neighboring microbes or host cells in the intestinal tract.

## Materials and methods

### Bacterial strains, culture conditions and reagents

*L*. *salivarius* (type strain #11741, American Type Culture Collection, Bethesda, MD) and *L*. *rhamnosus* (kind gift of M. Bailey, Nationwide Children’s Hospital, Columbus, OH) were maintained as frozen stocks. Reconfirmation of all strain identities prior to initiation of experiments was performed using MALDI-TOF (Bruker Inc., Billerica, MA, USA). The selection of *L*. *salivarius* as the prototypical microorganism to examine for the present study was based on its well-recognized ability to positively influence health and disease outcomes, such as those involving the microbiota-gut-brain axis, in a diverse group of animal species (for review see [21, 22]). As well, *L*. *rhamnosus* was utilized as a comparator strain as it has also been employed in similar studies [21]. Cultures were grown in Lactobacilli MRS broth (product #288130, Becton-Dickinson, Franklin Lakes, NJ, USA) from frozen stock the previous night in static culture in a 37°C humidified incubator. 4-(4-Diethylaminostyryl)-1-methylpyridinium iodide (ASP^+^; product #D-3418), 4-(4-(dimethylamino)phenyl)-1-methylpyridinium iodide (IDT307; product #SML0756, as well as other drugs and chemicals were obtained from Sigma-Aldrich (St. Louis, MO) and dissolved in double distilled water prior to use with the exception of carbonyl cyanide 3-chlorophenylhydrazone (CCCP, product #C2759) that was dissolved in ethanol with further dilution in IMDM to a final concentration in 0.5% ethanol in experiments. Decynium-22 (product #D4486) was purchased from TCI Chemicals (Portland, OR, USA) and was prepared in ethanol with further dilution in IMDM to a final concentration in 0.2% ethanol in experiments. All prepared solutions were sterile filtered prior to use and used at concentrations indicated in the text and figure legends.

### PMAT and OCT fluorescence-based assays

For measurement of PMAT-like transport, the IDT307 fluorophore-based Neurotransmitter Uptake Assay Kit (product #R8173, Molecular Devices, Sunnyvale, CA, USA) was used with modification. Similarly, for measurement of OCT-like transport, the ASP^+^ fluorophore was used in an assay identical to that for IDT307. These fluorophore-based assays, which we employed to examine putative neurotransmitter transporter activity in bacteria, employ the same approach utilized in mammalian cell culture to identify pharmaceutical compounds that target the activity of the biogenic amine transporters that are responsible for the uptake of dopamine, norepinephrine and serotonin [16]. The use of this assay design has proved instrumental in the identification and design of pharmaceutical compounds to treat depression and neurodegenerative diseases. To date, there are no reports that this assay design has been utilized with bacteria. In brief, the assay utilizes a fluorophore, either IDT307 or ASP^+^, in combination with a fluorescence masking agent, trypan blue. The fluorophores, which are actively taken into the cell via the PMAT (IDT307 fluorophore) or OCT (ASP^+^ fluorophore) transporters and thus mimic dopamine, norepinephrine and serotonin, are readily detectable by fluorescence using a bottom read detection mode. The presence of the masking agent completely quenches the fluorescence of the fluorophore only when it is adjacent to the fluorophore. Since the size of the masking agent, trypan blue, precludes its active uptake into the cell, only fluorophores that have been taken up by the cell via the transporter will be detected and that which is present in the extracellular fluid in the presence of the masking dye will be quenched and not detected. It is also important to note that the assay requires the use of a bottom read mode. The use of mammalian cells which are capable of attachment to the bottom of the well to form a monolayer is thus required for the read which was why bacterial biofilms were employed in this study.

As it is intended for studies with mammalian cells, the kit recommends the use of phenol red-free Hank’s Balanced Salt Solution (HBSS). For the results shown in this report, we substituted phenol red-free Iscove’s Modified Dulbecco’s Medium (IMDM; product #21056–023, Life Technologies, Carlsbad, CA, USA) for HBSS. Preliminary work showed that no transporter activity in bacterial biofilms could be detected with the use of HBSS (discussed further in Results section). IMDM was selected as it is a nutrient-rich, protein-free medium used in tissue culture that allows for robust *in vitro* growth of a wide variety of mammalian cell types in the absence of any protein additives common to most microbiology media. Its use has even extended to the culture of parasites [23, 24]. Similar tissue culture media, such as FluoroBrite DMEM (product #A1896701; Life Technologies), produced similar results to those obtained with IMDM (S1 Fig).

At the start of the assay, 0.2 ml of stationary phase overnight bacterial growth was inoculated into 9.8 ml of pre-warmed deMan, Rogosa and Sharpe (MRS), thoroughly mixed, and 0.2 ml aliquots added to individual wells of Corning flat and clear-bottomed 96 well microplates (product #3904, Corning, Inc., Corning, NY, USA). Plates were then placed into a 37°C humidified incubator. Following static incubation for either 6- or 24-hours, plates were removed from the incubator and the well supernatants were gently removed leaving the bacterial biofilm intact on the bottom surface of the individual clear plate wells. Subsequently, the PMAT-specific fluorophore IDT307 (Molecular Devices) or the OCT-specific fluorophore ASP^+^ (Sigma-Aldrich) dissolved in IMDM and pre-warmed to 37°C was gently added to appropriate wells (added along the well side so as not to disturb the biofilm). The plate was immediately placed into a BioTek Synergy H1 reader (Winooski, VT, USA) and measured using Gen5 software at the time intervals shown in the figures. All assays were done at 37°C unless otherwise noted and reads were performed as bottom fluorescence in relative fluorescence units (RTU). For IDT307, the chemical and masking dye were added in pre-measured amounts as supplied by the manufacturer and the amount of IMDM used as a diluent was the same as indicated for the use of HBSS. For ASP^+^, the dye and masking solution were prepared as previously reported [18] with the following minor modification: 100 μl of a 2 mg ASP^+^ stock solution per ml of distilled water was first added to 9.9 ml of IMDM and mixed, followed by the addition of 10 μl of a 10 mM Trypan blue solution. The excitation/emission wavelengths used for IDT307 and ASP^+^, respectively, were 440 nm/520 nm and 475 nm/609 nm.

As indicated above, IMDM was employed for the preparation of both the IDT307 and ASP^+^ fluorophores. This was in place of HBSS as the recommended fluorophore diluent since preliminary experiments utilizing HBSS for the preparation of IDT307 did not yield measurable levels of fluorophore uptake. The use of IMDM media likely provided a richer environment for maintaining bacterial function than HBSS, which is a simple minimal salt solution. Additionally, personal communication with Molecular Devices Technical Services staff confirmed that the use of HBSS had only been validated using eukaryotic cells that were stably transfected with human dopamine, NE or serotonin uptake 1 transporters. In this regard, fluorophore uptake in *L*. *salivarius* biofilms exceeded that observed by others in stably transfected eukaryotic cells.

### Growth assay

In order to examine if the fluorescent reporter dyes affected bacterial viability, increasing concentrations of IDT307 and ASP^+^ were added to cultures of *L*. *salivarius* that were prepared as 1:100 dilutions from overnight growth in MRS. *L*. *salivarius* diluted cultures with or without added fluorescent reporter dyes at concentrations indicated in S2A Fig (IDT307) and S2B Fig (ASP^+^) were added to triplicate wells of a 100-well microtiter plate and then incubated at 37°C with absorbance measured at 600 nm hourly for 24 hours using the automated Bioscreen C MBR instrument (Oy Growth Curves Ab Ltd., Helsinki, Finland) as previously described [25]. Treatment with IDT307 or ASP^+^ had no effect on bacterial viability since growth curves were identical to those observed in control cultures (S2A and S2B Fig, respectively).

## Results

**Fig 1:**
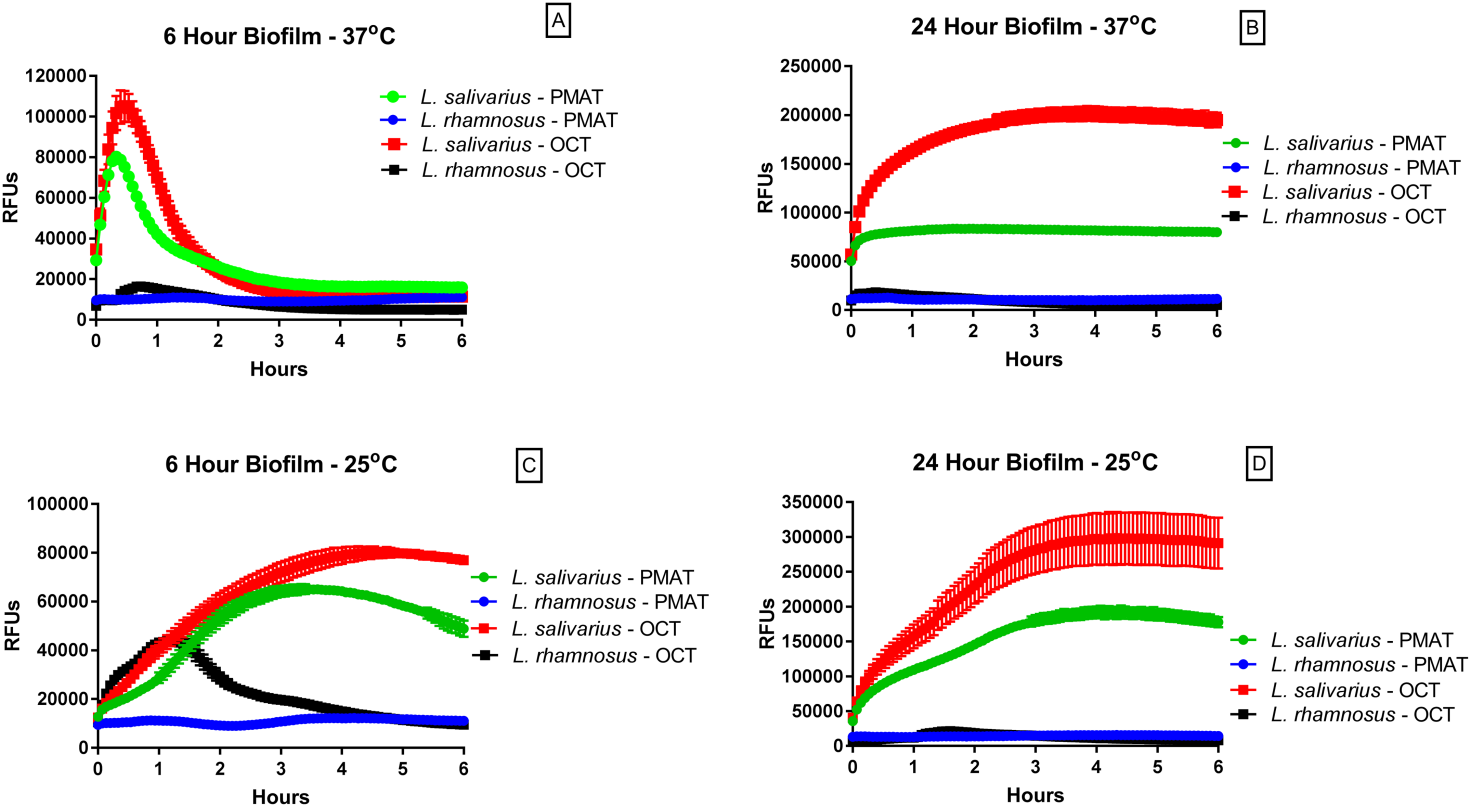
Uptake of transporter fluorophore reporters into *Lactobacillus* biofilms depends upon bacterial species and ambient temperature. Bacterial biofilms in flat clear-bottomed 96 well microplates were prepared as described in Materials and Methods. Following a 6 or 24 hour incubation of *L*. *salivarius* or *L*. *rhamnosus* to promote early or established biofilm formation, the indicated fluorophore, either IDT307 (probe for PMAT) or ASP^+^ (probe for OCTs), was added as per kit directions (IDT307) and a final concentration of 4 μM (ASP^+^), respectively, to appropriate wells and then immediately measured for fluorescence as described. For plates measured at 37°C (panels A and B), fluorophore solutions were pre-warmed and changes in relative fluorescence units (RFU) in each well of the plate were measured at 37°C over a 6 hour time interval. For plates measured at 25°C (panels C and D), the plate was removed from the incubator and placed on a bench-top surface for 1 hour to allow it to reach room temperature prior to fluorophore addition of the at room temperature. Following fluorophore addition, RFUs were measured in the plate at 25°C over a six hour time interval. Results represent mean ± S.E.M of quadruplicate wells and are representative of a minimum of 3 separate experiments. For some points, the S.E.M. error bars are obscured by the symbol.

In *L*. *salivarius* biofilms grown over a 6 or 24 hour period at 37°C, uptake of the IDT307 and ASP^+^ fluorescent probes over the succeeding 6 hour interval was rapid and attained maximum fluorescence intensity in < 60 min. Intracellular fluorescence decayed rapidly over the next 2–3 hours (Fig 1A and 1B). Biofilm thickness was significantly greater (*p*<0.0001) in *L*. *salivarius* cultures grown for 24 hours than those grown for 6 hours (S3 Fig). This phenomenon seemed to be selective for *L*. *salivarius* biofilms, as *L*. *rhamnosus* biofilms grown under the same conditions failed to take up either transporter probe (Fig 1A and 1B). In comparison to the 6-hour biofilms, fluorescence decay in *L*. *salivarius* biofilms was slower, suggesting that the probes had a longer residence time (> 6-hour) in the biofilms established over 24 hours (Fig 1A and 1B).

The uptake and decay of these fluorescent probes in both early and established *L*. *salivarius* biofilms were dependent upon ambient temperature as evidenced by slower increases in IDT307- and ASP^+^- associated fluorescence and extended time courses of fluorescence quenching in biofilms maintained at 25°C (Fig 1C and 1D). The mechanism underlying the decay of probe fluorescence is unknown, but could be attributed to fluoroprobe degradation in bacterial cells or quenching by bacterially-produced chemicals. Either potential mechanism could be affected by temperature.

**Fig 2:**
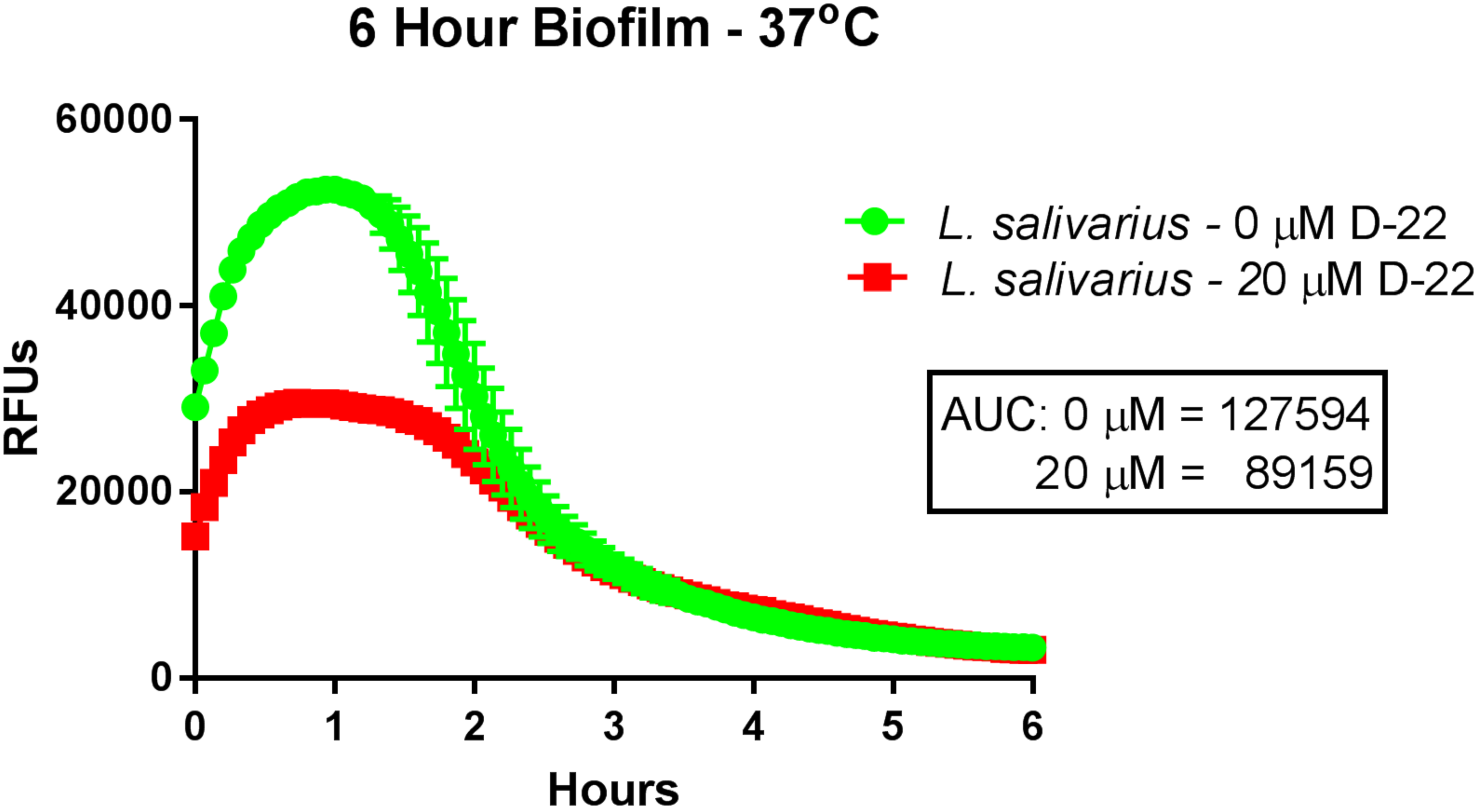
Effects of decynium-22, a cation transport inhibitor, on fluorophore uptake into *L*. *salivarius* biofilms. *L*. *salivarius* biofilms in flat clear-bottomed 96 well microplates were prepared as described in Materials and Methods. Following a 6 hour incubation of *L*. *salivarius* culture to form an early biofilm, the medium in each well was gently removed and 100 μl of a pre-warmed solution containing decynium-22 (D-22) was added to achieve a final media concentration of 20 μM (control wells contained the same solvent percentage as did the D-22 wells, see Materials and methods). The plate was then immediately placed back into a 37°C incubator for 30 minutes after which time it was removed and 100 μl of pre-warmed fluorophore was added to appropriate wells. The plate was then immediately placed into the fluorescence reader and measured at 37°C. Results represent mean ± S.E.M of triplicate wells and are representative of at least 2 separate experiments. For some points, the S.E.M. error bars are obscured by the symbol. AUC, area under the curve.

Decynium-22 is a potent, competitive substrate for low affinity, high capacity cation transporters, including PMAT and OCTs [26]. Peak increases in ASP^+^ uptake as determined graphically and through measurements of area under the curve (AUC) were blunted in early *L*. *salivarius* biofilms exposed to decynium-22 at a concentration of 20 μM (Fig 2A and 2B). At this concentration, decynium-22 was previously reported to block serotonin uptake through recombinant PMAT or OCTs expressed in HEK293 embryonic kidney cells [18].

**Fig 3:**
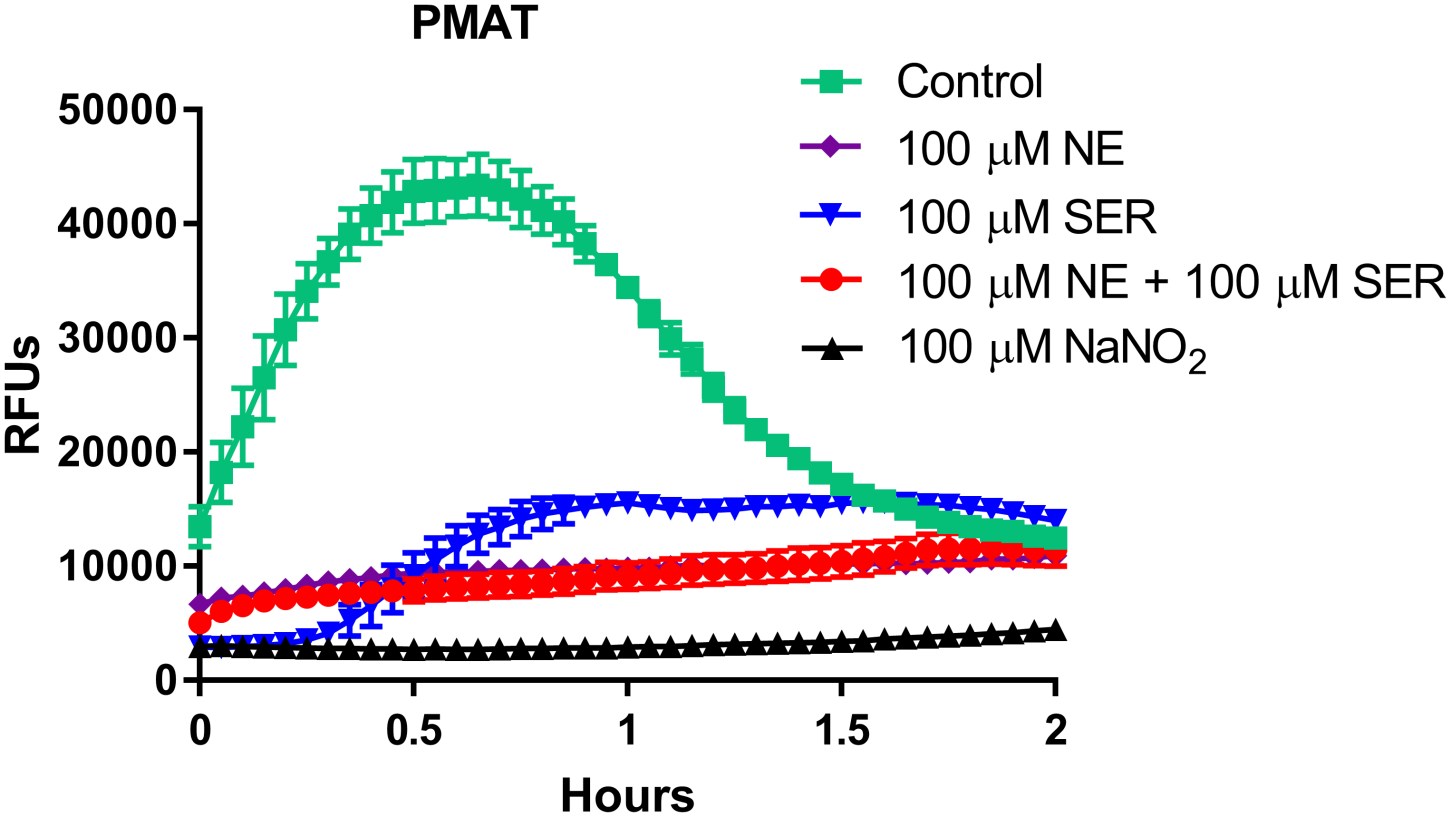
Ability of biogenic amine neurotransmitters and the metabolic inhibitor nitrate to inhibit IDT307 uptake into *L*. *salivarius* biofilms. *L*. *salivarius* biofilms in flat clear-bottomed 96 well microplates were prepared as described in Materials and Methods. Following 6 hours of incubation, the medium in each well was gently removed and 100 μl of a pre-warmed solution of the natural transporter substrates norepinephrine (NE) and serotonin (SER), alone or in equimolar combination, was added to appropriate wells to achieve a final biogenic amine concentration of 100 microM. The metabolic inhibitor sodium nitrite (NaNO_2_) was also added to appropriate wells to achieve a final concentration of 100 microM. The plate was then immediately placed back into a 37°C incubator for 30 minutes after which time it was removed and 100 μl of pre-warmed fluorophore as added to appropriate wells. The plate was then immediately placed into the fluorescence reader and measured at 37°C. Results represent mean ± S.E.M of triplicate wells and are representative of at least 2 separate experiments. The results for the NE only condition overlap those of the NE/SER condition. For some points, the S.E.M. error bars are obscured by the symbol.

Natural substrates for these transporters, including NE and serotonin, at a concentration of 100 μM markedly inhibited the uptake of IDT307-associated fluorescence into 6-hour *L*. *salivarius* biofilms (Fig 3). At an identical concentration, sodium nitrite also inhibited the rapid rise in IDT307-associated fluorescence (Fig 3). Among its many effects, nitrite ion inhibits active transport processes in aerobic and facultative anaerobic bacteria [27]. These data suggest that the *L*. *salivarius* biofilms express a PMAT uptake 2 active transport mechanism that is competitively inhibited by norepinephrine and serotonin.

**Fig 4:**
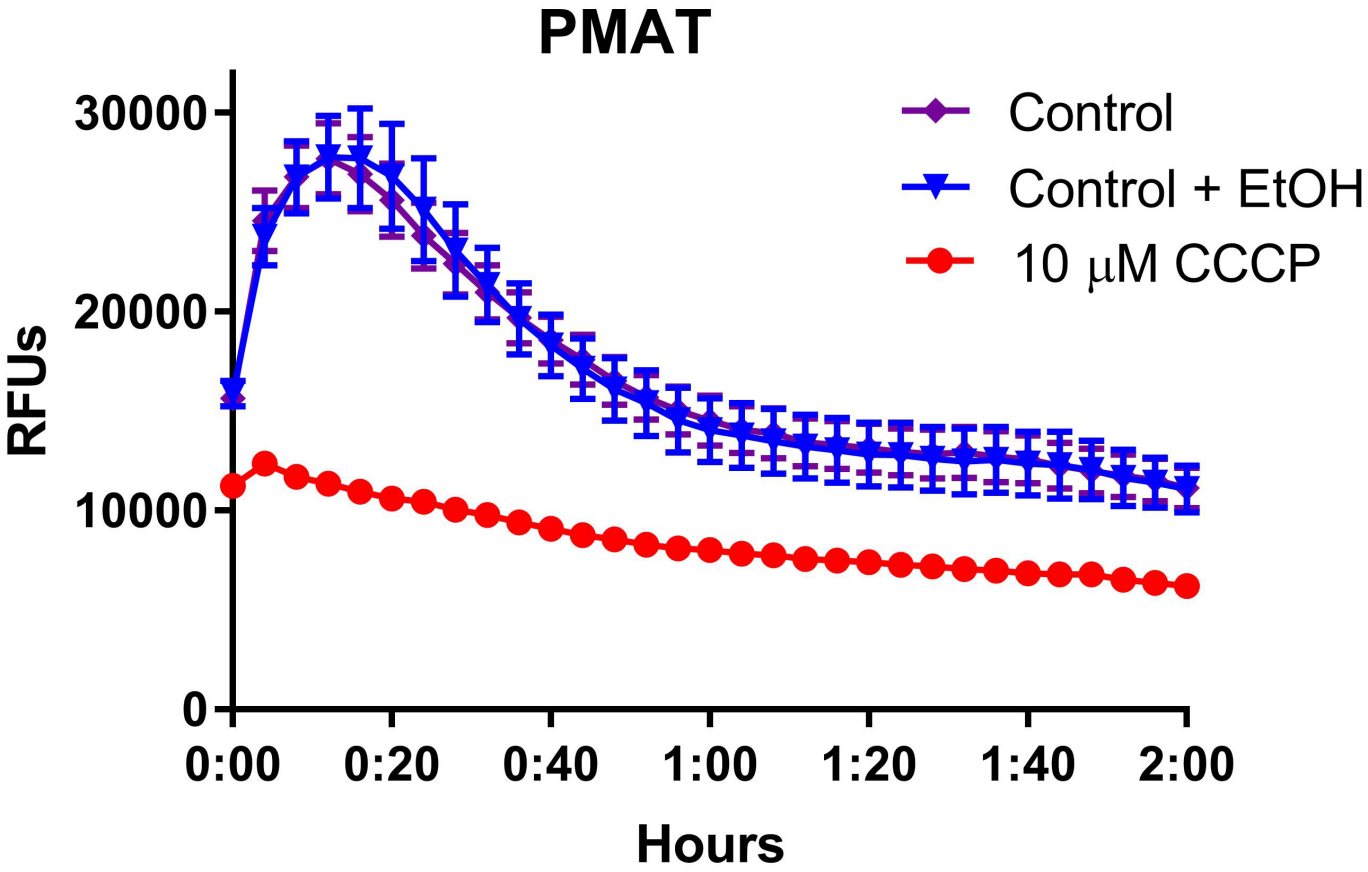
Ability of the protonophore CCCP to inhibit IDT307 uptake into *L*. *salivarius* biofilms. *L*. *salivarius* biofilms in flat clear-bottomed 96 well microplates were prepared as described in Materials and Methods. Following 24 hours of incubation, the medium in each well was gently removed and 100 μl of a pre-warmed solution of the proton ionophore carbonyl cyanide 3-chlorophenylhydrazone (CCCP) dissolved in IMDM (prepared as described in Materials and methods) or control IMDM with or without ethanol at an identical concentration present in CCCP-containing medium was added to appropriate wells. The plate was then immediately placed back into a 37°C incubator for 20 minutes after which time it was removed and 100 μl of pre-warmed IDT307 fluorophore was added to wells. The final concentration of CCCP was 10 μM following addition of IDT307 and the concentration of ethanol was 0.5%. The plate was then immediately placed into the fluorescence reader and measured at 37°C. Results represent mean ± S.E.M of triplicate wells. For some points, the S.E.M. error bars are obscured by the symbol.

This transport system appears to be driven by a proton-motive force, which is generally linked to ATP hydrolysis in several well-characterized, bacterial active transport and protein localization processes [28–30]. To test this hypothesis, we examined the effects of the protonophore carbonyl cyanide 3-chlorophenylhydrazone (CCCP), which dissipates plasma membrane proton gradients, to inhibit cellular uptake of IDT307. At a 10 micromolar concentration, CCCP completely inhibited the rise in IDT307-associated fluorescence compared to biofilms untreated with CCCP (Fig 4). This result suggests that fluorophore uptake is a proton-driven active transport process.

**Fig 5:**
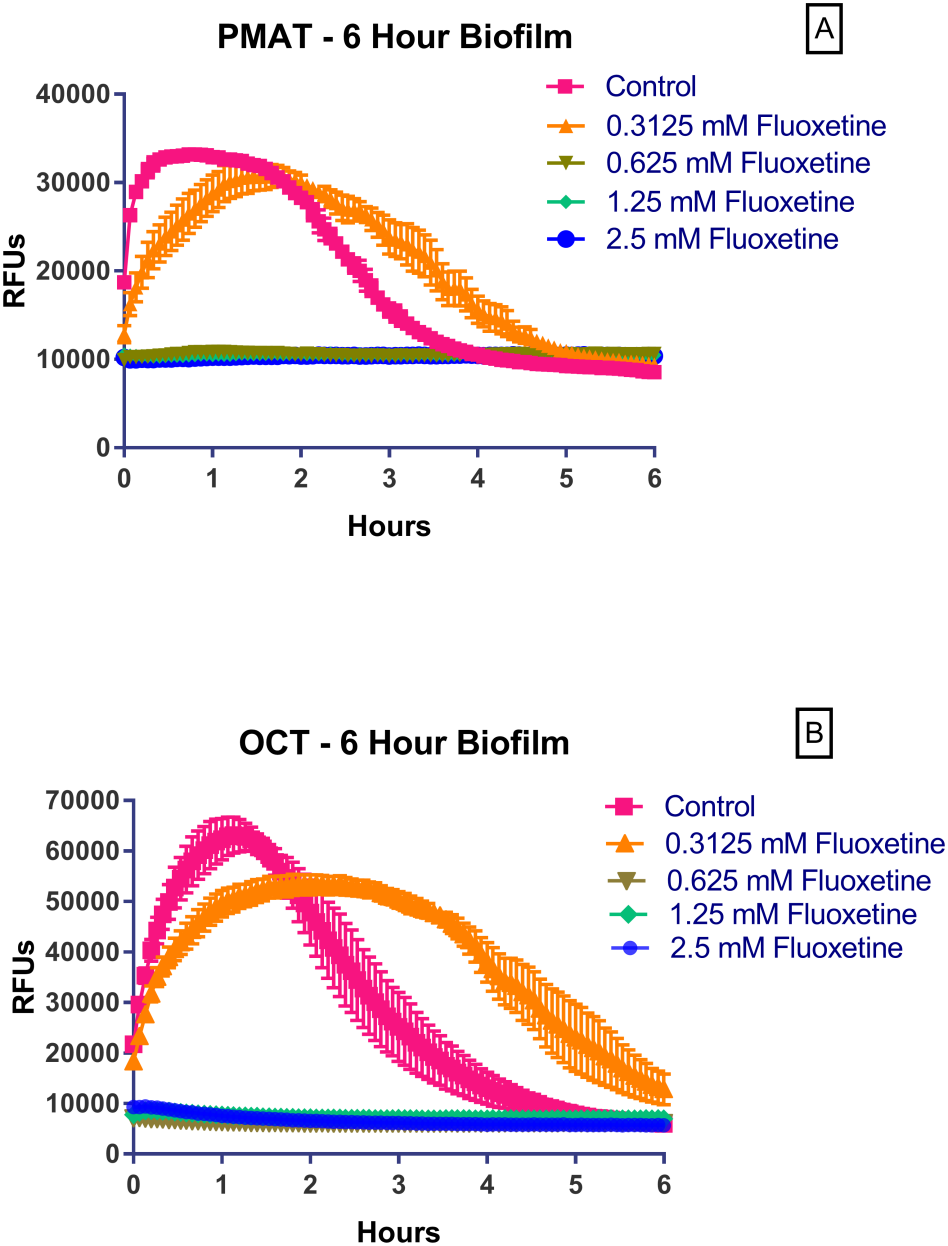
Concentration-dependent inhibition of IDT307 and ASP^+^ uptake by fluoxetine into early *L*. *salivarius* biofilms. *L*. *salivarius* biofilms in flat clear-bottomed 96 well microplates were prepared as described in Materials and Methods. Following 6 hours of incubation, the medium in each well was gently removed and 100 μl of a pre-warmed solution of fluoxetine dissolved in IMDM in the increasing concentrations shown in Fig 5 or IMDM alone was added to appropriate wells. The plate was then immediately placed back into a 37°C incubator for 30 minutes after which time it was removed and 100 μl of pre-warmed fluorophore was added to appropriate wells. The plate was then immediately placed into the fluorescence reader and measured at 37°C. Results represent mean ± S.E.M of triplicate wells and are representative of at least 2 separate experiments. For some points, the S.E.M. error bars are obscured by the symbol.

To further characterize SER uptake in early (6 hour) *L*. *salivarius* biofilms, we examined the effects of the selective serotonin reuptake inhibitor fluoxetine on uptake of IDT307 and ASP^+^. Fluoxetine inhibited uptake of either fluoroprobe into biofilms in a concentration-dependent fashion, with significant inhibition at 0.625 mM (Fig 5A and 5B). Similar results were obtained in established (24 hour) *L*. *salivarius* biofilms (S4 Fig).

## Discussion

In this study, we have provided evidence for the existence of bacterial PMAT- and OCT-like transporters that take up fluorescent probes previously determined to be specific for mammalian PMAT and OCTs. Fluorophore uptake appeared to be expressed in a species-dependent manner as it was detected in *L*. *salivarius*, but not *L*. *rhamnosus* biofilms. The uptake of IDT307 and ASP^+^, the respective fluorescent markers of PMAT and OCT-mediated uptake 2 transport was rapid, but less sustained in thinner (6-hour) biofilms than in thicker (24-hour) biofilms.

We attempted to conduct a preliminary characterization of the apparent cation transport function in *L*. *salivarius* biofilms by examining the dependence of fluorophore uptake on ambient temperature and nitrite-sensitive active transport. For each probe, the initial rise of fluorescence and the rate of fluorescence decay was slower at 25°C than at 37°C. However, peak fluorescence did not appear to be temperature-sensitive, suggesting that temperature affected uptake kinetics but not intracellular capacity for the fluorophore. In addition to its temperature sensitivity, uptake of the PMAT probe was abolished in biofilms pretreated with the metabolic inhibitor, sodium nitrite. As nitrite is a rather non-selective inhibitor of transport, we additionally examined the effects of the specific protonophore CCCP on IDT307 uptake in *L*. *salivarius* biofilms. As with nitrite ions, CCCP completely inhibited the uptake of this fluorophore into biofilms. These findings in sum suggest that uptake of these fluoroprobes into bacterial cells is an active transport process requiring cellular energy.

Mammalian OCTs are sensitive to the selective cation transport inhibitor decynium-22. This inhibitor was found to attenuate peak ASP^+^-associated fluorescence in *L*. *salivarius* biofilms, suggesting that an OCT-like transporter may be expressed in these bacteria. Moreover, the biogenic amines NE and serotonin similarly attenuated IDT307-associated uptake and peak fluorescence intensity in the biofilms, a result suggestive of competitive interactions with the fluoroprobes for cellular uptake. Finally, the serotonin reuptake inhibitor fluoxetine abolished fluorophore uptake into early and established *L*. *salivarius* biofilms at concentrations in the high micromolar range. In primary human hepatocytes and OCT-1 expressing HEK293 cells, fluoxetine completely inhibits OCT-1 mediated serotonin uptake at concentrations above 100 μM [31]. Antidepressant medications like fluoxetine have both salutary and adverse actions on gastrointestinal function, and it is possible that these effects may arise in part from drug-induced interference with the uptake of neuroactive amines by some members of the gut bacterial community [32].

The present results suggest that *Lactobacillus salivarius* and perhaps other species of bacteria residing in biofilms may be capable of taking up biogenic amines. Serotonin and dopamine concentrations measured in the mouse colonic lumen are approximately 5 nM and 2 nM, respectively, and the synthesis of these luminal substances is dependent upon gut bacteria [1, 33]. The bacterial uptake2 transporters could take up neurotransmitters in this concentration range and deploy them in bacterial interactions or affect the function of host cells. Their existence carries important implications for the ability of the host to interact with microorganisms through direct microbial-neurochemical interactions when excess neurochemicals may be secreted into the intestinal lumen, such as during periods of stress [34]. Bacterial analogs of biogenic amine transporters may contribute to the ability of the microbiota-gut-brain axis to influence brain function and ultimately behavior [35, 36].

## Conclusion

In demonstrating the existence of a biogenic amine transport system in bacteria, we examined members of the genus *Lactobacillus*. Although our selection of *Lactobacilli* spp. was based on the need to utilize biofilm-producing strains, it is also important to mention that the existence of an active, biogenic amine uptake mechanism may carry important implications for the use of these strains as probiotics. For example, *Lactobacilli* spp. have been used to treat conditions such as stress-induced alterations of gut permeability [37] and behavior [38], that have as a pathophysiological component the production of stress-related biogenic amines. It has yet to be determined if the function of *L*. *salivarius* or other probiotic bacterial species may be influenced by biogenic amine uptake via their expression of these amine transporters. Our present research is directed towards examining this possibility. Although the results describe the identification of these transporters in a biofilm-producing *Lactobacillus* species, the possible presence of such a transporter in other bacterial or even fungal genera, whether biofilm producers or not, remains open to examination [39]. A more widespread distribution in microbes may carry implications for understanding the recognized ability of the biogenic amines to influence the pathogenesis of a number of infectious diseases through microbial endocrinology interactions [40]. Although putative catecholamine “receptors” may mediate aspects of catecholamine-induced bacterial pathogenicity [6, 41–43], the existence of uptake 2-like cation transporters in these microorganisms may provide another avenue to investigate this phenomenon.

## Supporting information

S1 Fig Performance of the fluorescence-based transporter assay in two types of tissue culture medium. The IDT307 fluorophore Neurotransmitter Uptake Assay Kit was performed as described in Materials and Methods. Results show equal performance of the assay with *L*. *salivarius* biofilms regardless of whether IMDM or the FluoroBrite medium used.
(TIF)

S2 Fig Growth of *L*. *salivarius* in the presence of the fluorophores IDT307 and ASP^+^. The growth assay was performed as described in Materials and Methods. As shown, there was no effect of either IDT307 (PMAT transporter, A) or ASP^+^ (OCT transporter, B) on *L*. *salivarius* growth over a 24 hour incubation period.
(TIF)

S3 Fig Development of *L*. *salivarius* biofilm thickness. Biofilm optical density, as measured by absorbance of light at a wavelength of 600 nm, was significantly greater after 24 hours in culture than after 6 hours (p < 0.0001, paired t test).
(TIF)

S4 Fig Ability of selective serotonin reuptake inhibitor fluoxetine to influence uptake of the fluorophores IDT307 (PMAT transporter) and ASP^+^ (OCT transporter) in 24 hour *L*. *salivarius* biofilms. Assays were performed as described in Materials and Methods and demonstrate that fluoxetine inhibited the uptake of either fluorophore into biofilms in a concentration-dependent manner similar to that observed in the 6 hour biofilms shown in Fig 5A and 5B.
(TIF)

## Abbreviations

ASP^+^: (4-(4-(dimethylamino)styryl)-N-methylpyridinium iodide)
AUC: area under the curve
CCCP: carbonyl cyanide 3-chlorophenylhydrazone
D-22: decynium-22
IDT307: ((4-(4-(dimethylamino)phenyl)-1-methylpyridinium iodide)
IMDM: Iscove’s Modified Dulbecco’s Medium
NE: norepinephrine
OCT: organic cation transporter
PMAT: plasma membrane monoamine transporter
SER: serotonin
SLC: solute carrier transport protein
