# Supplementary figures and images for: Evidence for PMAT- and OCT-like biogenic amine transporters in a probiotic strain of Lactobacillus: Implications for interkingdom communication within the microbiota-gut-brain axis
Source: PLoS One. 2018 Jan 11;13(1):e0191037. doi: 10.1371/journal.pone.0191037 (PMC5764344; doi:10.1371/journal.pone.0191037)

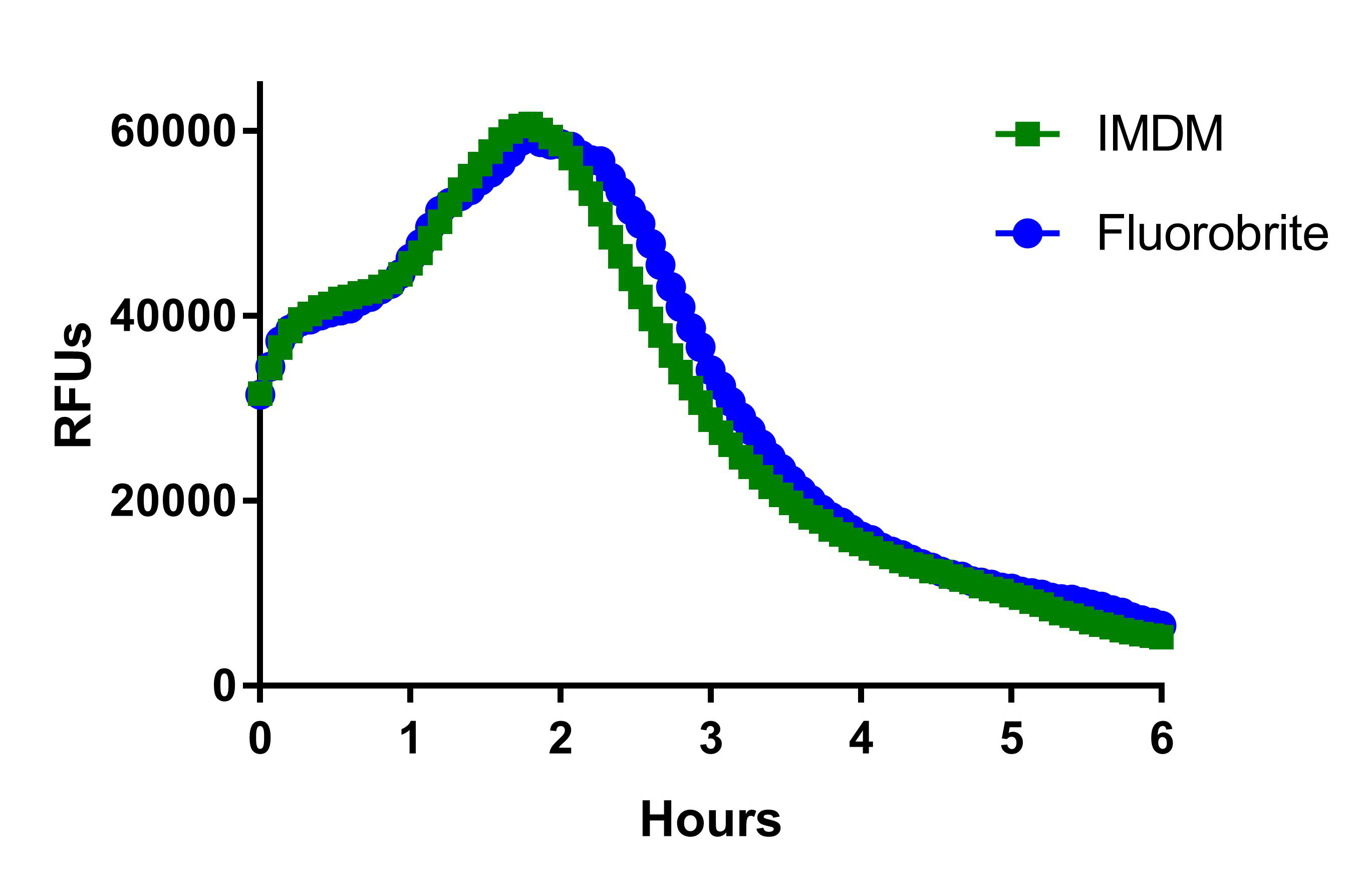

Supplement: S1 Fig — The IDT307 fluorophore Neurotransmitter Uptake Assay Kit was performed as described in Materials and Methods. Results show equal performance of the assay with L. salivarius biofilms regardless of whether IMDM or the FluoroBrite medium used. (TIF) [file pone.0191037.s001.tif]

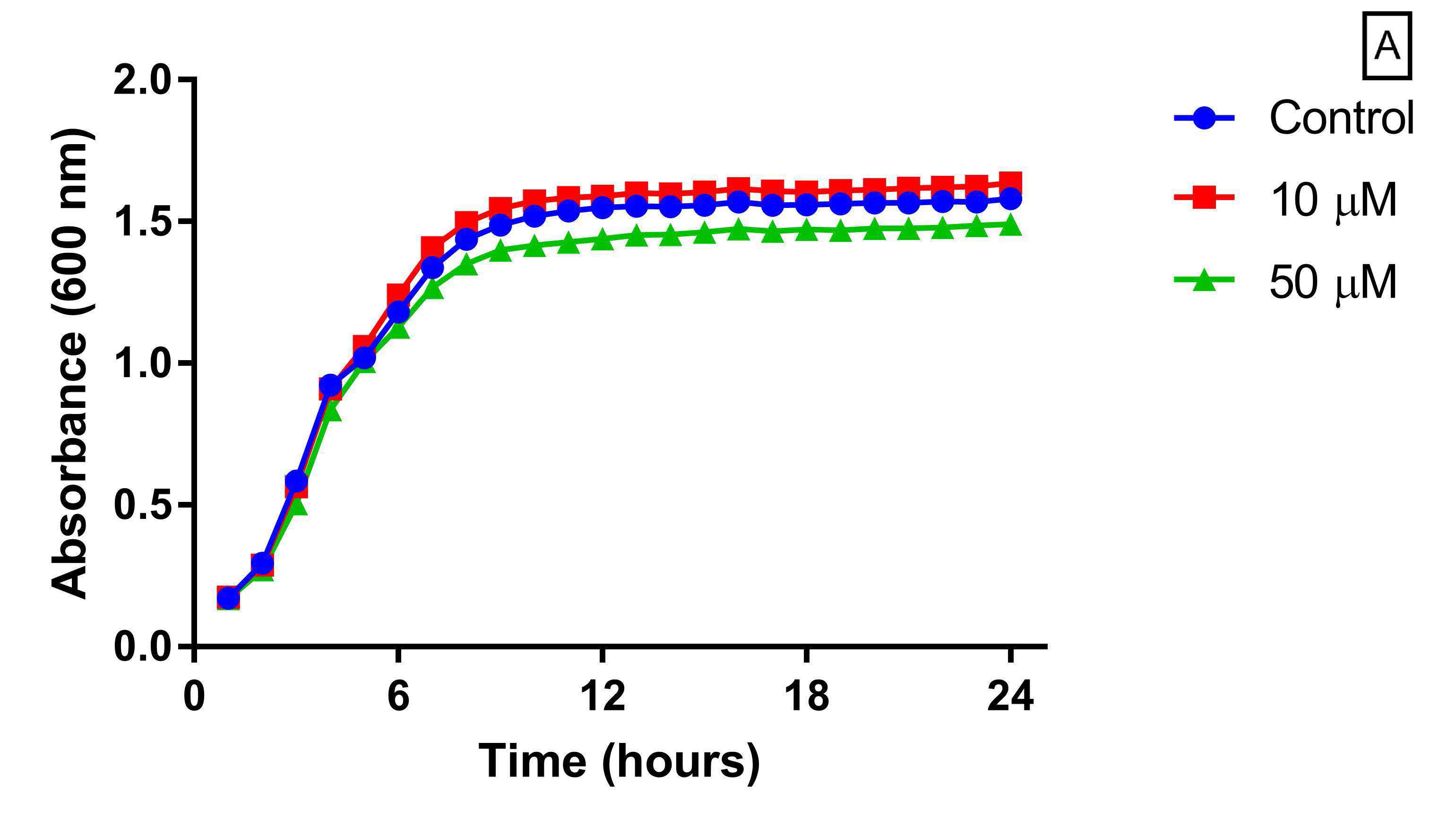

Supplement: S2 Fig — The growth assay was performed as described in Materials and Methods. As shown, there was no effect of either IDT307 (PMAT transporter, A) or ASP+ (OCT transporter, B) on L. salivarius growth over a 24 hour incubation period. (TIF) [file pone.0191037.s002.tif]

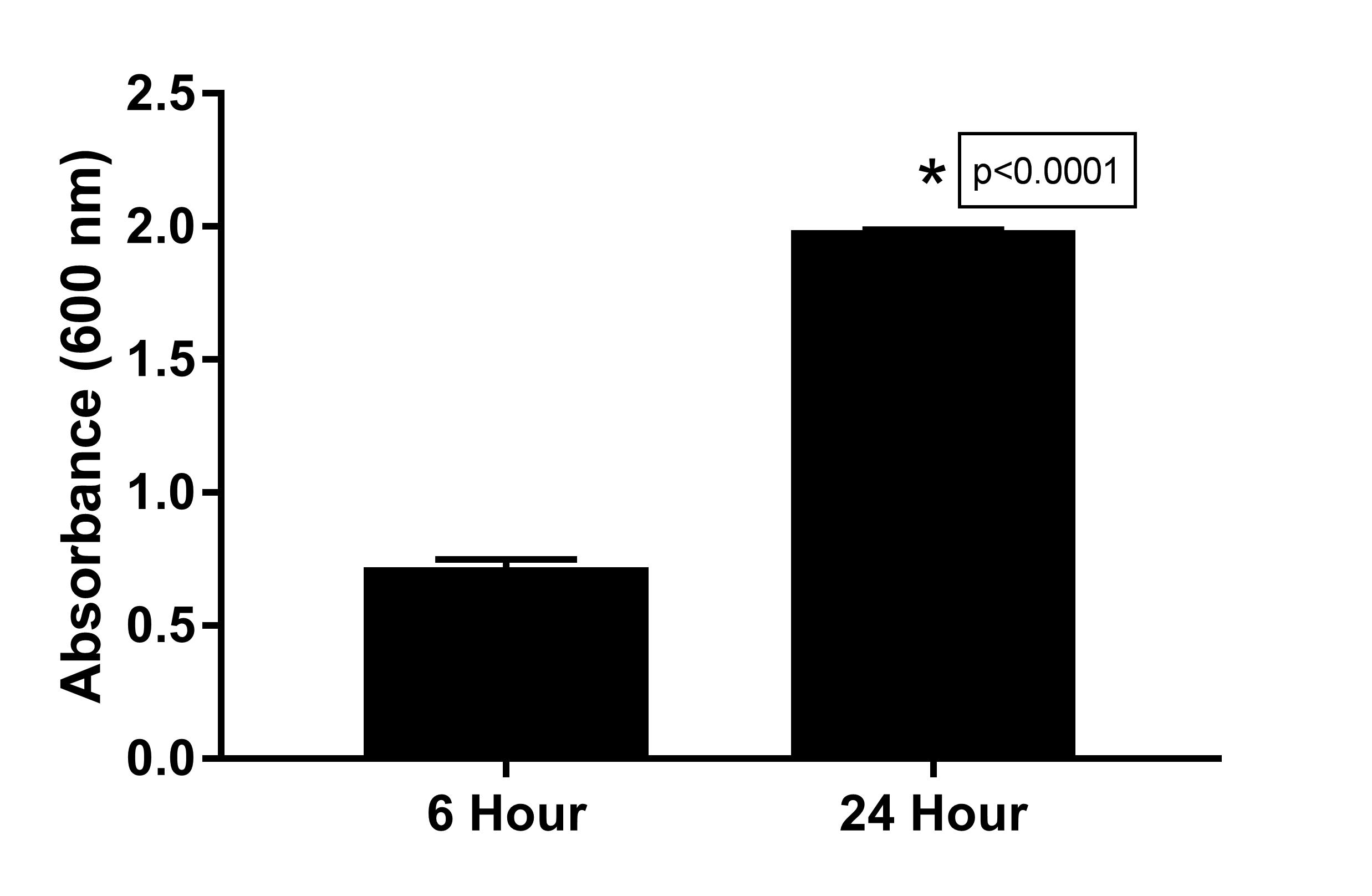

Supplement: S3 Fig — Biofilm optical density, as measured by absorbance of light at a wavelength of 600 nm, was significantly greater after 24 hours in culture than after 6 hours (p < 0.0001, paired t test). (TIF) [file pone.0191037.s003.tif]

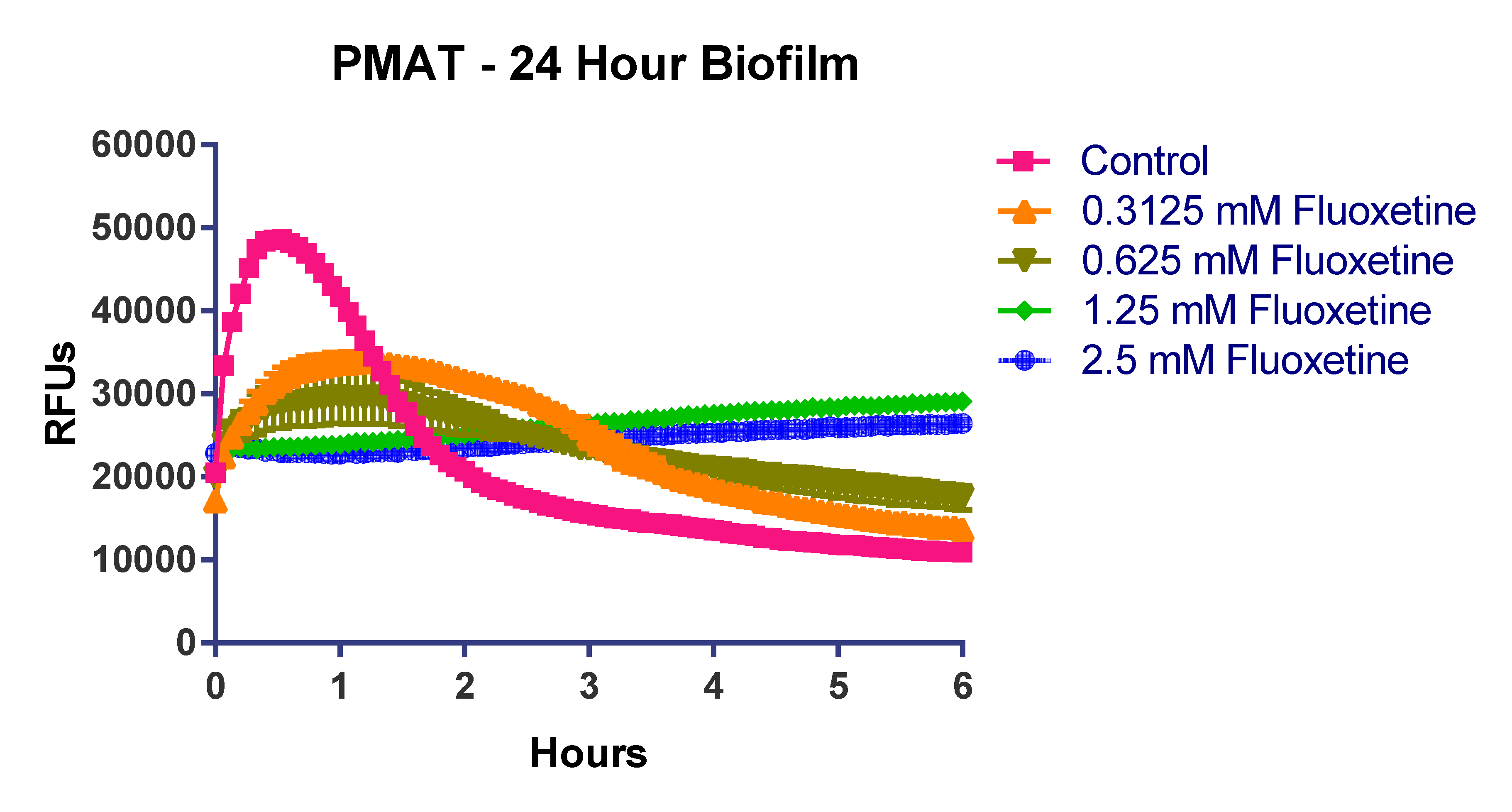

Supplement: S4 Fig — Assays were performed as described in Materials and Methods and demonstrate that fluoxetine inhibited the uptake of either fluorophore into biofilms in a concentration-dependent manner similar to that observed in the 6 hour biofilms shown in Fig 5A and 5B. (TIF) [file pone.0191037.s004.tif]
